# Initial symptoms and late complication in Lyme neuroborreliosis from the perspective of patients and relatives: a qualitative study

**DOI:** 10.1186/s12875-024-02624-w

**Published:** 2024-10-23

**Authors:** Anita Nymark, Lotte Huniche, Sigurdur Skarphedinsson, Helle Marie Christensen

**Affiliations:** 1 Department of Clinical Microbiology, Clinical Center for Emerging and Vector-Borne Infections, Odense University Hospital, J.B. Winsloews Vej 21, Entrance 245, 5000 Odense C, Denmark; 2 Department of Psychology, University of Southern Denmark, Odense, Denmark; 3 Clinical Center for Emerging and Vector-Borne Infections, Department of Infectious Diseases, Odense University Hospital, Odense, Denmark; 4 Department of Clinical Research, University of Southern Denmark, Odense, Denmark; 5 Department of Respiratory Medicine, Odense University Hospital, Odense, Denmark

**Keywords:** Vector-borne diseases, Lyme neuroborreliosis, Patient participation, Qualitative research, Patient perspective, Focus group

## Abstract

**Background:**

Lyme borreliosis is by far the most common vector-borne infection in Western Europe. The most severe manifestation of Lyme borreliosis is Lyme neuroborreliosis (LNB). In LNB symptoms vary from mild to severe and may include late complications that involve both physical and/or neurocognitive constraints. An estimated 25–28% of the LNB population suffers from late complications. This study investigates patient and relative perspectives on everyday life with LNB symptoms, diagnosis, and treatment to identify areas for improvement of healthcare.

**Methods:**

A focus group was conducted at Odense University Hospital, Denmark. The focus group comprised 16 participants, nine patients diagnosed with LNB who had been treated at the Clinical Center for Emerging and Vector-borne Infections, and seven relatives of the patients’ choice. The focus group lasted 2 ½ hours and was audio recorded as well as documented in field notes.

**Results:**

Data analysis was grounded in the conceptual framework of critical psychology and resulted in three main themes: (1) Burden of LNB symptoms in everyday life, (2) A break in the conduct of everyday life caused by LNB and (3) Need for transparent pathways to specialist knowledge.

**Conclusions:**

Before diagnosis and treatment, each patient reported varying degrees of non-treatable pain, and cognitive and/or musculoskeletal symptoms. Visible physical symptoms were rare. All patients had experienced that their bodily symptoms remained unaddressed throughout numerous encounters with the healthcare system. The course of LNB comes with a break in patients’ everyday lives and self-understandings affecting their ability to work and manage everyday activities. Patients and relatives strongly recommend a specialised LNB clinic.

## Introduction

Lyme neuroborreliosis (LNB) is an infection in the central nervous system (CNS). Symptoms vary from mild to severe and include late complications that involve both physical and neurocognitive constraints [1]. Over the years, awareness of late complications has increased among clinicians and has been reported in the literature, and often referred to as Post-Treatment Lyme Disease (PTLD). An estimated 25–28% of the LNB population suffers from late complications following diagnosis and treatment [2, 3].

In 2018, ECDS’s surveillance programme was updated and Lyme neuroborreliosis was specifically added to the program. Indicating that LNB is of increasing significance [4]. In Denmark, LNB is included in a list of diseases at the statutory Danish notification system for infectious diseases (DNSID). Recent studies have revealed that the incidence in Denmark has been underestimated [5]. A previous Danish survey showed that, from the onset of neurological symptoms to diagnosis and treatment, there was an average delay of 24 days, resulting in a frequency of 28% of long-term sequelae, increasing to 44% among patients with treatment delay > 30 days. The overall delay was found associated with an increased likelihood of late complications [6]. The same study revealed that the majority of patients diagnosed with LNB had had several prior contacts with the healthcare system that had not resulted in a confirmed diagnosis of LNB or effective treatment. Research on this topic has mainly taken a quantitative approach; however, clinical experience has shown that essential knowledge is to be gained from patient’s illness trajectories, their journey from onset of symptoms to LNB diagnosis, including late complications. This study was conducted at the Clinical Centre for Emerging and Vector-borne Infections (CCEVI), Odense University Hospital (CCEVI), and investigated the perspectives of patients with confirmed LNB diagnosis according to current guidelines [7]. Patients were encouraged to bring a relative of their own choice to the focus group. The aim was to gain a broader insight into the course of the disease as experienced in everyday life, including symptoms, diagnosis, treatment, and complications over time. Based on these insights, we aimed to point to possibilities and constraints of existing clinical practice and to suggest strategies for organisational improvement.

## Methods

The focus group study was grounded in the conceptual framework and methodology of critical psychological practice research (CPPR). CPPR is a qualitative research approach akin to other forms of action research, but specifically grounded in the theoretical framework of critical psychology [8–10]. The cornerstones of the CPPR approach are: (1) a close link between developing theories and qualifying professional practice, (2) a focus on participants’ concerns in personal lives, and/or in professional practice from their first-person perspectives, (3) involvement of and collaboration with participants as co-researchers, e.g., patients, relatives, health professionals, policymakers, etc. Thus, the CPPR approach comes with the aim of closely tying knowledge production to the development of personal, professional, and organisational practices [8, 10]. Fundamental to this endeavour is the exploration of research participants’ perspectives on concrete problematics, e.g. in everyday lives with illness or in clinical practice.

### Design

#### Setting

One large focus group was conducted at Odense University Hospital, with the first author as facilitator and last author as co-facilitator. First author holds an MSc in Health Science and is a registered nurse with 20 years of clinical experience within the field of infectious diseases, particularly vector-borne diseases. First author has conducted clinical research based on both quantitative and qualitative methods. As a clinician at CCEVI the first author and facilitator of the focus group was known to the participating patients and relatives.

#### Data collection

Patients were recruited from the CCEVI database according to the following criteria: (1) Diagnosed with LNB according to the “Danish National Guideline”, (2) Having terminated treatment with antibiotics 1 to 1½ years previously, (3) Ability to and interest in communicating their entire LNB illness trajectory. Fifteen patients were invited by e-mail to take part in the focus group and encouraged to bring a relative of their choice. Six patients declined for reasons related to working hours and lack of energy. Relatives were included for two main reasons. First, relatives are commonly important participants in the everyday lives and illness trajectories of patients. According to CPPR human beings dialectically interact with others in structures of social practice and should therefore be understood, not in isolation from social contexts, but as part of the everyday contexts in which they participate [11]. Secondly, clinical observations has shown that the triad between patient, relative and a health care professional creates space for new insights and realisations that can play a significant part in the course of LNB and patients’ conduct of everyday life [11].

During the focus group participants were seated in a horseshoe-shape, thus enabling all participants to see each other and the facilitators. A guide for conducting the focus group was developed and applied [12, 13]. Topics included: (1) Early manifestations of symptoms/signs of illness, (2) The course of LNB, (3) Relatives’ perspectives on early manifestations and everyday life, (4) Challenges, including shortcomings of the healthcare system, (5) Current state of health and way of life, (6) Suggestions for how clinical practice could improve [14]. The purpose of the study and the focus group agenda was iterated to confirm written consent. The participants were requested to speak one at the time for the purpose of audibility. Pens and paper were placed in front of the participants, who were asked to write down any remarks or questions and raise a hand to indicate that they wanted to contribute. The co-facilitator (last author) acted as an observer and took field notes. Participants and their relatives had been invited for a two-hour session which was extended 2 ½ hours. The decision to extend the time frame was due in part to the large number of participants, but more importantly, to the wealth of participants’ contributions and their expressions of gratitude at this opportunity to voice their experiences, concerns, and needs. The focus group was audio recorded and transcribed verbatim using the NCH software [15]. Field notes were analysed along with the transcript [16].

#### Ethics

The study was approved by the Danish Data Protection Agency Journal nr. 16/31,743. All participants signed an informed consent form before entering the study. Names and identifying characteristics of patients and relatives have been changed or omitted.

#### Analytical framework

For the analysis of patients’ and relatives’ perspectives, we drew on the theoretical framework of CPPR [8, 17]. This framework is founded in historical dialectical materialism, which is an approach to understanding the nature of human beings as fundamentally social and societal. When a person’s physiological, emotional, or cognitive functioning is affected by illness, the ability to act and participate will also be affected. Changes occur in how everyday life is conducted and in how the persons can participate in everyday social and societal activities. Hence, altered functioning because of illness requires adjustment in everyday life. In particular, the concept of *conduct of everyday life* was central to our analysis [9, 10]. *Conduct of everyday life* points to the “active performance of persons with the intention of connecting various activities into a coherent whole” [11]. This active performance and coordination of living everyday life with others in and across social practices comes with *possibilities* and *constraints* [11]. Staging conduct of everyday life as a central point of reference in the analysis of patients’ and relatives’ perspectives on LNB afforded a focus on altered functioning, concrete symptoms, difficulties, and ways of managing, as experienced in the context of everyday life and in relation to healthcare over time. When human beings experience a shift in their action possibilities that requires a response, the shift or break affects the person’s conduct of everyday life. According to Ole Dreier, such shifts or *breaks* also affect a person’s *self-understanding*, given that it does not hinge on internal psychic processes, but emerges from the person’s conduct of everyday life in specific circumstances and with others [11, 18]. Moreover, the concept of conduct of everyday life directs attention to everyday *routines*, which are necessary to uphold everyday life. Routines are commonly taken for granted and left unnoticed but become visible when day-to-day living is challenged by a shift or break in the structure of the conduct of everyday life. Routinised activities maintain the structure and content of everyday living, conserve energy and form the foundation for engaging in other forms of activities in everyday life [11, 18]. Moreover, the conduct of everyday life is marked by transitions or timeouts, such as, for example, the transition between jobs, going on vacation, falling ill, being hospitalised, etc. Some shifts, breaks, transitions or timeouts are planned and some occur without notice [11, 18]. After a timeout, such as a weekend stay away, to everyday routines are resumed much as before, whereas breaks due to severe symptoms of disease call for major adjustments or simply impose changes on everyday routines [11, 18]. Prolonged periods of severe symptoms, particularly if they reduce everyday functioning and have not yet been medically explained, can fundamentally alter the basis of a person’s everyday conduct of life and self-understanding [18].

#### Data analysis

The audio recording of the focus group was transcribed verbatim using NCH-Software. The transcript was read several times along with the field notes by the first and last author to identify meaningful units. The working procedure was inspired by Braun and Clarke’s six phases of analysis: (1) Familiarising with data, (2) Generating initial codes, (3) Searching for themes, (4) Reviewing themes, (5) Defining and naming themes and (6) Producing the report [19]. The conceptual framework of critical psychology offers an understanding of *persons conducting their everyday lives in concrete contexts that are part of wider societal practices* (e.g., clinical practice) *and overall structures* (e.g., the organisation of the healthcare system). Critical psychology has an analytical orientation towards *conditions*,* meanings*,* and reasons* that guides the overall analytic process towards the investigation of how actual *conditions* in everyday life (including those of illness, late complications, and treatment) *mean* something to individuals (e.g., being in pain, not being able to sustain a working life), and how personal actions are *reasoned* from a first-person perspective [10]. Based on this analytical framework, we aimed to gain knowledge about patients’ conduct of everyday life and personal reasons for acting in particular ways in specific situations, e.g., when faced with impaired functionality, challenged self-identity, and when struggling to have symptoms recognised and treated. When investigating a concrete scene in a local social practice from a first-person perspective, it is important to recognise that the scene is part of, and affected by other social practices as social practices hang together in peoples’ everyday lives. Hence, to understand a social practice in a specific location, it is necessary to investigate how the local situation hangs together or is linked with other social practices [20].

## Results

The focus group comprised 16 participants, nine patients (six women and three men) and seven relatives of the patients’ choice, including six spouses (one woman and five men) and an adult daughter. Patients were 38–68 years of age. Patients reported a span of two to 52 weeks from the onset of symptoms to the start of treatment Table 1.

**Table 1:** Participants

Invited patients (female/male)	7/8
Number of participated patients (female/male)	9 (6/3)
Number of relatives (female/male)	7 (2/5)
Patients married/single	8/1
Patients age range (mean/median)	38-68 years (54/59)
Weeks from onset of symptoms to treatment (range)	2/52 weeks (mean 12 weeks)

Three main themes emerged from the analysis: (1) Burden of LNB symptoms in everyday life, (2) A break in everyday life with LNB. (3) Need for transparent pathways to specialist knowledge Table 2.

**Table 2:** Focus group discussion themes

1 theme	2 theme	3 theme
Burden of LNB symptoms in everyday life	A *break* in the conduct of everyday life with LNB	Need for transparent pathways to specialist knowledge

### Theme 1: Burden of LNB symptoms in everyday life

A tick bite that leads to LNB is associated with specific symptoms in the group of patients who took part in the focus group. Table 3 provides an overview of symptomatology divided into primary symptoms and late complications. Late complications centred on neurocognitive and physical symptoms, and work-life.

**Table 3:** LNB symptoms and work status

**Symptoms before diagnosis**	**Number**
Radicular pain at night	9
Flu like symptoms	3
Musculoskeletal pain	8
Pain and restless legs	5
Sensory bodily disturbance	4
Fatigue	5
Short time memory problems	4
Facial nerve palsy	4
Erythema migrans	1
**Late complication (1 year after antibiotic treatment**	**Number**
Fatigue	6
Reduced executive performance^a^	3
Short time memory problems	3
Musculoskeletal pain	1
Sensory bodily disturbance	1
**Work status (1 year after antibiotic treatment)**	**Number**
Unchanged	2
Unable to work	1
Early retirement benefit	1
Working less hours	2
Retire	2
Changed job	1

^a^Measured by Symbol Digit Modality test (SDMT)

#### Symptomatology before antibiotic treatment

Pain was the most common symptom before initiation of antibiotic treatment, as reported by the focus group participants, particularly radicular and sensory pain. Poor night sleep due to pain was emphasised by six of the nine patients:

“I slept for a maximum of 1½ hours and then I woke up with unbearable pain in my lower back and spent the rest of the night in a chair. This went on for several weeks” [Female D].

Painkillers did not completely solve the pain issues for any of the patients and some experienced severe side effects:

“I ended up with pain-plaster (morphine) and it only got me to feel strange in the head” [Female C].

Flu-like symptoms (shivering, sore muscles, headache but without actual fever) were also relatively common among the participants.

“During the summer I sensed a feeling of the flu, just without fever” [Female A].

Cognitive challenges were expressed by all patients to varying degrees, such as concentration problems and consequently loss of skills:

“Normally I work full time but this tick bite made me unable to work more than 15 hours pr. week” [Male I].

Visible physical symptoms were more rarely observed. Some patients suffered from facial paresis and spoke of it as a ‘good symptom’ because it ended their journey and resolved their search for a diagnosis:

“When I got the paresis in the left side of my face, my GP concluded at last that I had to be admitted for further examination, he spoke of infarct or a hemorrhagic bleeding, but never Lyme neuroborreliosis” [Female B].

#### Symptomatology 12 months after antibiotic treatment

Apart from one, all participants were struggling to varying degrees with neurocognitive and other physiological late complications 12 months after antibiotic treatment.

Brain fatigue was reported as the most common neurocognitive symptom. Although it was not captured by patients in medical definitions, it was experienced during everyday activities. The following is an extract of reported brain fatigue situations:

“I was not able to finish cooking a meal” [Female B],

“I was struggling to find the right words” [Female F],

“After 3 hours at work my brain would stand still, as if you had pressed a mute button” [Male H],

“In the middle of the grocery shop I couldn’t remember what to buy and was overwhelmed by all the noise and light – when I got out I cried and had to sit in my car for some while before I was ready to drive home without any groceries” [Female C],

“I had to sleep at least two hours after lunch to function just a little bit with my family in the early evening, and still went to bed at nine o’clock” [Male I],

“My everyday life is turned around – I practically do nothing and feel tired and exhausted most of the day” [Female D].

This kind of brain fatigue was different from any kind of fatigue that patients had ever experienced. It left everyday life unpredictable and barely manageable, as patients were never sure of where or when exhaustion would set in. Radicular and sensory symptoms also added to the difficulties patients experienced in attempting to manage everyday life as they used to. One patient [Female D] decided to retire at the age of 62 because, among other neurocognitive symptoms, she also described significant sensory disturbance in her fingers and arms and weak muscles in her legs. Another, [Male H], changed jobs to day shifts rather than nights because of the limitations brought on by LNB. Overall, symptoms were experienced as having a profound impact on the everyday lives and wellbeing of both patients and their relatives.

### Theme 2: A break in the conduct of everyday life with LNB

The focus group participants conveyed a sense of how everyday life had been massively impacted by bodily symptoms, impaired functioning, and the search for an explanation. To these patients, the experience of LNB was life-changing. Phenomena such as ‘flu-like symptoms’, including fever, sensitive skin, sore muscles, headaches, and lack of all-round well-being, were well known to patients, but only as phenomena that would disappear spontaneously with little or no medical treatment. ”It felt like influenza but in a very special way” [Female A].

At the outset of LNB, patients understood such phenomena as a temporary disturbance in everyday life that induced uncertainty.

“It was some trouble, both I and my wife were very anxious about my health” [Male I].

However, as time passed and symptoms persevered or worsened, everyday life was disrupted in more fundamental ways, to the point where patients had to cut down on everyday activities, change jobs, reduce working hours, or give up work entirely.

“My experience of LNB made me rethink my job situation and I now no longer work at night, it’s so much better for my health” [Male H].

And

“I still cannot work because of all the symptoms LNB caused me” [Female F].

Everyday life was also vastly impacted by the seemingly futile search for explanations, medical recognition, treatment, and relief. The consequences of the disease itself, along with the lack of a diagnosis and assistance from the healthcare system, thus constituted a break in the conduct of everyday life, prior routines, and self-understandings. Particularly challenging were the numerous contacts with the healthcare system, e.g., General Practitioners (GP), chiropractors, and staff at emergency departments, without getting any closer to a resolution or to finding productive ways forward. One participant [Female E] reported as many as 12 contacts over a period of five weeks, mainly consultations with the GP. In searching for answers, all patients consulted the internet. This often left them more anxious, frustrated, and worried, rather than reassured, to the extent that one patient stated:

“Burn down the internet” [Male H].

A couple of relatives stated how difficult it was to witness their spouses clearly not being themselves, suffering, and needing help, and further having to act as spokespersons in relation to the healthcare system that had to be pushed into providing their services [Female D and E’s s relative]. Even after receiving the LNB diagnosis, several of the participants struggled because there was a lack of specific rehabilitation programmes and because they were considered able to work and not eligible for sickness benefits. None of the patients had a sense of being taken care of sufficiently during the initial debut of LNB:

“Not even the neurologist was aware of what could be the matter with me… I am surprised how little they know or maybe it was only this one who did not know anything, but then again, he was a doctor and isn´t he supposed to know? [Male I].

This data, coupled with clinical experiences from the CCEVI, supports the assertion that left untreated in the preliminary diagnosis process, LNB can lead to severe late complications and breaks in everyday life, including the loss of the ability to work [6].

The patients expressed both new and unaddressed bodily symptoms and revealed a search for ways to overcome this new bodily status. The symptoms changed their action possibilities and led to a break or change in their conduct of everyday life. They expressed mostly constraints and struggles due to symptoms but also made great efforts to address the symptoms, to find a way to be diagnosed correctly and to receive proper treatment and care. The patients described their contacts with the healthcare system, mostly the GP or other health professionals, as a search for associations between their symptoms and a diagnosis, to adapt their bodily symptoms into their conduct of everyday life and thus also their self-understanding. To understand patients’ reactions, critical psychology can address a way to understand their symptoms, and how the course of LNB hangs together, by identifying pathways, seeking to understand how changes in a local context are affected by other social and societal contexts, such as care provision and the overall structure of healthcare in which they take place [20]. One relative [I] stated that her husband was seen by five different physicians at the hospital and none of them suspected *Borrelia*. Another finding was that none of the patients were diagnosed at their first appointment with a physician. Often, several visits to different kinds of healthcare professionals were needed before receiving the diagnosis.

### Theme 3: need for transparent pathways to specialist knowledge

A need was expressed for a healthcare facility where patients could turn to with their daily challenges in dealing with symptoms. All patients expressed their gratitude to the clinic CCEVI:

“I would have been lost without you [the CCEVI]” [Female E].

The patients and relatives voiced their thoughts and ideas about such a centre even before they were asked.

Patients considered it highly relevant to be referred to a specialist clinic with various treatment options, for several reasons. The availability of experts in vector-borne infections improved the patient’s chances of having their symptoms and difficulties in everyday life recognised. The patients also considered it to be more likely to be diagnosed and offered relevant treatment faster. Recognition by specialised healthcare professionals induced a sense of relief that they had, finally, ended up in the right place in the healthcare system. The majority of the patients agreed that it took time to comprehend and find ways of conducting everyday life with the impairments caused by a LNB. Both patients and relatives requested better connection through their journey from symptoms to diagnosis and treatment. The fact that all the patients represented some symptoms of LNB, but not all patients represented the same range of symptoms could have some impact on patients’ experiences of constraints and possibilities in managing everyday life with LNB. They presented themselves as a group with overlapping symptoms. Only one of the patients remembered that he had had a tick bite during the period, where he remained undiagnosed and his erythema migrans was not considered severe enough to warrant treatment. The rest did not recall a tick bite before having the symptoms.

Some of the patients reported [Male I’s relative, Female B, C, D and E] having consulted several physicians before the suspicion of LNB was raised either by a GP or by a physician at the hospital. A specialised context responsible for examining, diagnosing, informing, educating, and treating tick bites in general, and LNB in particular, was considered important by the participants. All patients agreed that they would have valued a pamphlet or some kind of written material, including a description of a typical course of LNB. Furthermore, late complications ought to be described to forewarn and prepare patients for what to expect and to allow time to adjust to the situation and consider action possibilities. One relative was the adult child of a LNB patient and specifically requested a pamphlet or video with facts about LNB addressed to children. This relative also suggested gathering a group of children to investigate what children need and how to meet such needs. This kind of investigation should be age-sensitive.

The patients expressed several reflections on establishing a patient group facilitated by former patients and nested at the CCEVI. An online site connected to the hospital or directly to the clinic was suggested, and some patients pointed to Facebook as a way of making and staying in contact. The patients also suggested that CCEVI should be engaged in educating other healthcare professionals and in developing visual materials for that purpose, e.g., examples of erythema migrans, facial palsy, etc. The GPs should be first in line to learn about *Borrelia*, as they see the largest number of patients and serve as gatekeepers to other parts of the Danish healthcare system.

One relative pointed out that it would be helpful if the clinic briefed on pension funds that pay out a sum to people who receive a LNB diagnosis.

## Discussion

### Limitation and strength of the method

The size of the focus group was slightly larger than the literature recommends [16]. An obvious limitation was that participants may not have had the opportunity to voice all relevant symptoms and concerns. The option to divide participants into two focus groups was considered but dismissed based on various limitations to the kinds of interactions possible. The decision to proceed with a large focus group was related to the concept of conduct of everyday life. As persons live together with others in and across social practices, we need to gain knowledge from both patients’ and their relatives’ first-person perspectives, to understand how patients conduct their everyday lives with LNB. To ensure that everyone was heard each participant was given the opportunity to speak their mind at a certain time during the focus group. For the rest of the time, participants spoke in turn after a show of hands. Six female and three male patients participated in the study. It is possible that it could have strengthened the results if gender was represented more evenly. However, the results showed that patients shared the same kinds of struggles, regardless of gender. The age range was 38–68 median of 59, reflecting the general adult LNB population [6, 21]. The results showed that the focus group provided participants with a rare opportunity to be heard and have their struggles recognised, which also contributed to a highly productive group dynamic. Thus, the data was rich and highly informative. The meeting was extended at the participants’ request. Both patients and relatives, regardless of gender reasoned that they wanted their experiences to be of use to future patients, and several participants offered to sign up for other research projects, which can be regarded as an expression of a desire to contribute to strengthening the field.

The facilitator of the focus group was part of the outpatient clinic, which meant that the participating patients had previously met the facilitator in a hospital setting. This could conceivably have influenced the results. All participants had completed their treatment and follow-up in CCEVI before the focus group was conducted. To decrease the impact of participants’ connection to CCEVI, It was voiced by the facilitator at the focus group, that all participants would be anonymised in further analysis and publication of the results. Furthermore, the facilitators reiterated that the participants could withdraw without any further explanation, in accordance with the overall premise that research is based on voluntariness.

### Organisational development

Over the years, awareness of late complications in LNB has increased among clinicians and has been reported in the literature. The Danish healthcare system is organised with GPs as gatekeepers; they most frequently refer patients for diagnosis at a public hospital. After an observation and examination period, which can be up to 48 h, the physician from the emergency services or the emergency department of a hospital refers patients to a specialised department for further diagnosis and treatment or discharges them into GP care [22]. In Denmark, rehabilitation is the responsibility of local health authorities and is provided by a range of different healthcare services. The patients’ perspectives in this study demonstrated that any number of social practices were activated in different locations throughout the course of LNB. Better management strategies for early detection and treatment are needed. The results highlighted that there was a need for collaboration to link the patients’ needs for recognition, diagnosis, treatment, and rehabilitation. A transparent pathway can add predictability to the patient’s situation, aid their self-understanding, and provide a basis for re-establishing routines in everyday life. Self-understanding refers to one’s ability to understand and incorporate into one’s self-awareness the bodily symptoms, reduced functionality, and altered everyday life due to LNB. For healthcare professionals to contribute in this way to a better self-understanding can support subsequent changes in the conduct of everyday life and adjust to new situations.

### Relevance to clinical practice

Based on the results from the focus group and existing literature, clinical practice can arguably be developed to minimise late complications. Developing clinical guidelines can assist healthcare professionals in various contexts to recognise *Borrelia* and/or have the patient referred to a relevant specialist or clinic. It would be an advantage to include a specific question in a clinical symptomatology guideline about patients’ sleep quality, as six out of nine patients in the current study said they had poor sleep at night and that they frequently had to get out of bed. LNB is not a common infection but often comes with the complexity of multiple issues around health and well-being issues. Therefore, treatment and follow-up should be the responsibility of specialists in vector-borne infections. Specialised *Borrelia* clinics are recommendable and, in addition to specialist physicians and nurses, would benefit from the services of a neuropsychologist, a medical social worker, and a physiotherapist with expert knowledge of their field and borreliosis.

## Conclusion

Patients experienced late complications in the form of neurocognitive and/or musculoskeletal symptoms. Pain was the most common symptom before initiation of antibiotic treatment, particularly radicular pain and sensory disturbance. Flulike symptoms were also relatively common among the participants. Cognitive challenges were expressed by each patient to varying degrees, such as concentration problems and, consequently, loss of skills. Visible physical symptoms were rarely observed. Another finding was that late complications were a constraint associated with a break in the patient’s life trajectory and conduct of everyday life. This break was associated with impaired bodily functioning, challenges to patients’ self-understanding, and the prolonged search for an explanation. To these patients, LNB was experienced as profoundly life-changing.

The results from the focus group suggest that further symptomatology research is needed, e.g., radicular pain that is not curable with any kind of painkillers could be associated with LNB. Furthermore, investigations into short-term and long-term symptomatology and consequences for the conduct of everyday life can shed light on the course of LNB, to show how late complications correlate with delay from the start of symptoms until diagnosis [6].

Finally, the study points to the necessity of additional investigation regarding transparent pathways between cross-sectional participants of all kinds who are involved in the care of LNB patients.

## Data Availability

Anonymised data are available from the corresponding author upon reasonable request. Empiric field data and transcriptions are available for all authors.

## Abbreviations

LNB: Lyme neuroborreliosis
CPPR: Critical Psychological Practice Research
ECDC: European Center for Disease Prevention and Control
DNSID: Danish Notification System for Infectious Diseases
CCEVI: Clinical Centre of Emerging and Vector-borne Infections
